# Analysis of complications associated with peripherally inserted central venous catheters. Prospective observational study

**DOI:** 10.15649/cuidarte.3352

**Published:** 2024-12-27

**Authors:** Marta Ferraz-Torres, Ana Diez-Revilla, Ruth Plaza-Unzue, Maria Inés Corcuera-Martinez

**Affiliations:** 1 PhD, MMCE, RN, University Hospital of Navarra, Navarra, Spain. Associate professor, Department of Health Sciences, Public University of Navarre, E-mail: marta.ferraz@unavarra.esResearcher, IdiSNA, Navarra Institute for Health Research Universidad de Navarra Department of Health Sciences Public University of Navarre Navarra Spain marta.ferraz@unavarra.es; 2 RN, University Hospital of Navarra, Navarra, Spain. E-mail: ana.diez.revilla@navarra.es Universidad de Navarra University Hospital of Navarra Navarra Spain ana.diez.revilla@navarra.es; 3 RN, University Hospital of Navarra, Navarra, Spain. E-mail: ruth.plaza.unzue@navarra.es Universidad de Navarra University Hospital of Navarra Navarra Spain ruth.plaza.unzue@navarra.es; 4 RN, Hospital University Hospital of Navarra, Navarra, Spain. E-mail: ines.corcuera.martinez@navarra.es Universidad de Navarra University Hospital of Navarra Navarra Spain ines.corcuera.martinez@navarra.es

**Keywords:** Vascular Access, Nursing Team, Oncology, Hematology, Treatment, Acceso Vascular, Equipo de Enfermería, Oncología, Hematología, Tratamiento, Acesso Vascular, Equipe de Enfermagem, Oncologia, Hematologia, Tratamento

## Abstract

**Introduction:**

Vascular access teams often use guidelines or algorithms to determine the most appropriate vascular access device based on the patient's condition and the substance to be infused. These guidelines are intended to help identify the most qualified personnel for device insertion, but few studies collect information on the performance of these units.

**Objective:**

This study aims to identify the evolution and complication rate of peripherally inserted central catheters (PICCs) in patients requiring vascular access.

**Materials and Methods:**

A prospective observational study was conducted over three years. Continuous variables with normal distribution were compared using Student's t-test. Nonparametrically distributed variables were analyzed with the Mann-Whitney U test. For categorical variables, the two-tailed chi-square or Fisher's exact test was used. Regression analysis was performed for the dependent variable of complications.

**Results:**

Of the PICCs inserted, 61.99% (566) were in patients receiving oncologic treatment, with a mean dwell time of 136±127.51 days. PICCs inserted in hematologic patients had a mean dwell time of 144±141.3 days (p=0.438). The most frequent complications were accidental removal (3.50%, 32, OR 0.581), thrombosis (3%, 27, OR 0.752), and central line-associated bloodstream infection (CLABSI) (2.10%, 19, OR, 0.113).

**Discussion:**

Complications related to PICCs were infrequent, with thrombosis being the most prevalent. Accidental removal was also frequent, a complication not thoroughly analyzed in other studies.

**Conclusions:**

PICC insertion and management by vascular access teams enables units to achieve a low complication rate in onco-hematological patients.

## Resumen.

**Introducción:**

Los equipos de acceso vascular suelen utilizar guías o algoritmos para determinar el dispositivo de acceso vascular más adecuado según el paciente y la sustancia que se va a administrar. Se supone que las guías permiten al personal identificar a la persona más calificada para insertar el catéter, pero pocos estudios recogen información sobre el desempeño de estas unidades.

**Objetivo:**

Este estudio busca identificar la evolución y la tasa de complicaciones de los catéteres centrales de inserción periférica (PICC) en pacientes que requieren acceso vascular.

**Materiales y Métodos:**

Estudio observacional prospectivo de tres años de duración. Las variables continuas con distribución normal se compararon mediante la prueba t de Student. Las variables distribuidas con distribución no paramétrica se analizaron con la prueba U de Mann-Whitney. Para las variables categóricas se utilizó la prueba chi cuadrado de dos colas o la prueba exacta de Fisher. Se realizó análisis de regresión para la variable dependiente “complicaciones”.

**Resultados:**

El 61,99% (566) de los PICC fueron insertados en pacientes con tratamiento oncológico y una duración media de 136±127,51 días. Los PICC usados en pacienteshematológicostuvieronunaduraciónmediade144±141,3días(p=0,438). Las complicaciones más frecuentes fueron la remoción accidental (3,50%, 32, OR 0,581), trombosis (3%, 27, OR 0,752) bacteriemia asociada al catéter venoso central (2,10%, 19, OR, 0,113).

**Discusión:**

Las complicaciones relacionadas con los PICC fueron poco frecuentes, siendo la principal la trombosis. Las remociones accidentales fueron frecuentes, una complicación que no ha sido suficientemente analizada en otros estudios.

**Conclusiones:**

La inserción y manejo de los PICC por un equipo de acceso vascular permite a las unidades lograr una baja tasa de complicaciones en pacientes oncohematológicos.

## Resumo.

**Introdução:**

As equipes de acesso vascular geralmente usam diretrizes ou algoritmos para determinar o dispositivo de acesso vascular mais adequado de acordo com o tipo de paciente e a substância a ser infundida. Supõe-se que as diretrizes permitam que a equipe identifique a pessoa mais qualificada para a inserção do acesso, mas poucos estudos coletam informações sobre o desempenho dessa unidade.

**Objetivo:**

O presente estudo tem como objetivo reconhecer a evolução e a taxa de complicações de cateteres centrais de inserção periférica em pacientes com acesso vascular.

**Materiais e Métodos:**

Estudo observacional prospectivo durante três anos. As variáveis contínuas com distribuição normal foram comparadas usando o teste t de Student. As variáveis com distribuição não paramétrica foram analisadas com o teste de Mann-Whitney. Para variáveis categóricas, foi usado o teste de qui-quadrado bicaudal ou o teste exato de Fisher. A análise de regressão foi realizada para a variável dependente de complicações.

**Resultados:**

61,99% (566) dos cateteres centrais de acesso periférico foram inseridos em pacientes tratados com abordagem oncológica, com uma vida útil média de 136±127,51 dias. Aqueles inseridos em pacientes hematológicos tiveram uma meia-vida de 144±141,3 dias (p=0,438). As complicações mais frequentes foram saídas acidentais (3,50%, 32, OR 0,581), trombose (3%, 27, OR 0,752) e infecção da corrente sanguínea associada à linha central (2,10%, 19, OD 0,113).

**Discussão:**

As complicações dos cateteres venosos centrais de acesso periférico foram baixas, sendo a trombose a principal complicação. As saídas acidentais foram frequentes, uma complicação não analisada em outros estudos.

**Conclusões:**

A inserção e o gerenciamento desses cateteres por uma equipe de terapia intravenosa permitem que as unidades alcancem um baixo índice de complicações em pacientes onco-hematológicos.

## Introduction

The Peripherally Inserted Central Catheter (PICC) has become a mainstay in the care of hospitalized patients, providing long-term, high-quality intravenous access thanks to a low risk of complications^1^. However, implementing and managing a PICC is complex and involves many aspects to control. The insertion technique requires choosing the best vascular option for the patient according to the substance(s) to be infused, choosing the most appropriate PICC gauge according to the size of the target vessel, and executing the procedure with precision. In addition, managing a PICC requires nursing professionals who are specifically trained in the care and maintenance of this device and capable of handling any complications that may arise from its use^2^^, ^^5^. One option to improve the PICC insertion and management process is establishing a Vascular Access Team (VAT) within hospitals.

A VAT is a group of healthcare professionals whose primary role is to evaluate, insert, manage, monitor, and analyze data from their services, solve clinical problems, and, when necessary, remove central venous catheters^6^. Institutions with aVAT are more likely to use tools, guidelines, or algorithms to determine the most appropriate vascular access device^7^ according to the substance(s) to be infused and to identify who is the best-qualified personnel for inserting a specific vascular device, with the aim of achieving better patient outcomes^2^. Few studies have focused on quantifying the direct impact of the presence of a highly trained VAT on reducing complications and improving the quality of vascular access care with PICCs^4^^, ^^8^^, ^^9^. Some studies have reported thrombotic complication rates of 1-4% (3.1 to 4.6 per 1,000 catheter/days) ^9^^, ^^12^, 4.62% among cancer patients, and 0.3-1.48 per 1,000 catheter/days in hematological patients^13^. Central line-associated bloodstream infection (CLABSI) rates have been reported at 2.1%^12^ to 9.4%^14^. However, few studies have prospectively collected such data over long time periods, which may result in lower-quality results.

Therefore, our work aims to measure the overall complication rate associated with the use of long term PICCs in oncological and hematological patients and the direct effect of VAT-led insertion and management of these devices.

## Materials and Methods

A prospective observational study was conducted by recording and monitoring long-term PICCs inserted in all adult patients (>18 years old) admitted to our center’s hematology and oncology units, with catheter placements performed by the VAT between January 2018 and January 2021.

The sample size was calculated based on an estimate with a type I error of 5% and a type II error of 10% (90% of power). According to these parameters, 923 catheters were needed for the study sample. Of the 923 catheters recorded over the 3 years, 913 were deemed valid for inclusion in the study.

Data were collected through direct patient follow-up, evaluation, and electronic recording, with prior patient consent and information.

The sociodemographic variables included the date of admission, sex, medical specialty at admission, and data related to the PICC insertion (date of insertion and removal, site of insertion, punctuated vein, catheter gauge, number of lumens, and insertion length). As an outcome variable, the main reasons for catheter removal were tracked and categorized as completion of the treatment without complications, death, discharge, or interhospital transfer, or the presence of complications, such as accidental dislodgement, CLABSI, Medical Adhesive-Related Skin Injury (MARSI), phlebitis, obstruction, thrombosis, and other, secondary complications.

### Definitions

*Venous thromboembolism* is defined as either (1) the development of a symptomatic thrombosed vessel (partial or complete) at the PICC site, diagnosed via ultrasound, or (2) symptomatic deep vein thrombosis (DVT), as described by the trial investigator^3^.

CLABSI refers to primary bacteremia or fungemia with at least one positive blood culture from a peripheral vein with no other identifiable source for infection other than the PICC, plus one of the following must be present: a positive semiquantitative (> 15 colony-forming units [cfu]) or a quantitative device culture (> 103 cfu), with the same organism (species and antibiogram) isolated from both the PICC and blood; two blood cultures (one from the PICC hub and one from a peripheral vein), meeting PICC-related bloodstream infection criteria for quantitative blood cultures (three fold greater colony count of growth for the same organism as from the peripheral blood); or meeting the criteria for differential time to positivity (growth of the same microbe from hub-drawn blood at least 2 hours before growth from the peripheral blood)^3^.

*Occlusion* is a complete blockage of the PICC lumen(s), including fibrin sheath and medication precipitate^3^. This includes aspiration and infusion occlusion, and occlusions that resolve with tissue plasminogen activator, and intraluminal thrombosis or fibrin sheath as described by the trial investigator.

*MARSI* refers to superficial layers of skin removed by medical adhesive, in which erythema and/or other manifestations of skin trauma or reaction, such as vesicles, bulla, skin erosion, or skin tears, persist for longer than 30 minutes after adhesive removal^15^.

### Statistical analysis

Continuous variables with a normal distribution were compared using Student's t-test, while non- parametrically distributed variables were analyzed with the Mann-Whitney U test. Categorical variables were assessed using the chi-square test or two-tailed Fisher's exact test. Continuous variables data are expressed as means and standard deviations (SD) or medians and interquartile ranges, alongside percentages with a 95% confidence interval (CI). A two-tailed test was used to determine statistical significance, which was accepted at p<0.05. Regression analysis was conducted for the dependent variable ‘complications’ using the patient’s department as a selection variable and including the demographic variables with statistically significant values (p<0.05) as covariates. The regression model was previously evaluated using the R-squared metric and mean squared error^16^. Statistical analysis was performed using SPSS version 26. A p-value of less than 0.05 was considered significant. All collected data is freely accessible and available for consultation in Mendeley Data^17^.

### Ethics

This study was conducted in accordance with the Declaration of Helsinki. Participants were informed, and informed consent was requested prior to data collection. Anonymity and data protection were guaranteed during the study. This study was approved by the Ethics Committee of Navarra with code (PI_2021/75).

## Results

In total, 60.35% (551) of the PICCs were inserted in women and 39.64% (362) in men. Sixty-two percent (566) were placed in patients receiving medical oncology care, with a mean PICC dwell time of 136 days (SD 127.51). For hematological patients, mean PICC dwell time was 144 (SD 141) (p=0.438) (Table 1).

**Table 1:** Demographic data of cancer and hematological patients who had a catheter placed by the VAT

		Medical specialty		
	Total %(913)	Hematology %(n=344)	Oncology %(n=569)	p-value
Sex				<0.001**
Male	39.64 (362)	20.79 (190)	30.21 (172)	
Female	60.35 (551)	16.88 (154)	43.22 (397)	
PICC mean dwell time (days)*	138 (134)	144 ±141	131 (118)	0.438**
Vein				0.959***
Basilic	85.43 (780)	32.18 (293)	53.42 (487)	
Brachial	14.01 (128)	5.41 (49)	8.69 (79)	
Cephalic	0.54 (5)	0.22 (2)	0.28 (3)	
Catheter gauge				<0.001***
3FR	0.21 (2)	0.11 (1)	0.11 (1)	
4FR	72.17 (659)	14.28 (131)	58.89 (528)	
5FR	25.84 (236)	22.51 (206)	3.31 (30)	
6FR	0.54 (5)	0.49 (5)	0	
No. of lumens				<0.001**
1	73.60 (672)	14.50 (132)	59.11 (540)	
2	25.41 (232)	22.19 (203)	3.21 (29)	
3	0.87 (8)	0.89 (8)		
No. of punctures				0.995**
1	86.85 (793)	34.51 (298)	57.16 (495)	
2	6.90 (63)	2.83 (24)	4.50 (39)	
3	0.65 (6)	0.21 (2)	0.47 (4)	
4	0.32 (3)	0.11 (1)	0.21 (2)	

*X (SD) ** Student’s t ***Chi-square

The most common insertion site was the right arm (71.70%, 654), typically using the basilic vein, with a mean insertion length of 40 cm (3.91). Complete insertion of the device, after measurement of the length to be inserted, was achieved in 73.32% (669) of the cases. In 73.58% (672) of cases, single-lumen catheters were used, and 59.11% (540) of the devices were placed in cancer patients. In 86.89% (793) of the cases, a single puncture was sufficient for proper device placement, while two punctures were needed in 6.90% (63) of cases. Among all procedures performed with ultrasound guidance, which accounted for 90.62% (827), the insertion and tip location technique utilized intracavitary electrocardiography.

**Table 2:** Dependent variables of the study by patient's treatment specialty

		Medical specialty		
Reason for PICC removal	Total % (913)	Hematology %(n=344)	Oncology %(n=569)	p-value
Hospital discharge	1.45 (13)	0.93 (8)	0.52 (5)	0.134*
Death	23.70 (216)	8.35 (76)	15.35 (140)	0.387
Replacement	0.70 (6)	0.59 (59)	0.11 (1)	0.05*
Treatment completion	63.01 (575)	22.71 (207)	40.31 (368)	0.172
Recorded complication				
Thrombosis	2.95 (27)	1.65 (15)	1.30 (12)	0.05
Accidental catheter removal	3.50 (32)	1.50 (14)	2.01 (20)	0.726
Obstruction	0.22 (2)	0.11 (1)	0.11 (1)	1*
MARSI	1.90 (18)	0.72 (7)	1.18 (11)	1*
Extravasation	0.11 (1)	0	0.11 (1)	0.437*
CLABSI	2.13 (19)	1.13 (10)	1 (9)	0.174
Catheter rupture	0.11 (1)	0.11 (1)	0	0.799*
Upper extremity paresthesia	0.11 (1)	0	0.11 (1)	1*

Chi-square *Yates’ continuity correction for values with a minimum expected count <5

Accidental removal was the most common complication, occurring 3.50% (32) of the cases, followed by thrombosis (2.95%, 27) and CLABSI (2.13%, 19). Accidental catheter removal occurred mainly among cancer patients (2%, 20), while thrombosis was the most prevalent complication among hematological patients (1.65%, 15) (Table 2). According to the binary logistic regression analysis, hematology patients were more likely to develop thrombosis than cancer patients (Table 2). The regression analysis also identified the number of lumens of the device as the main risk factor for some of these types of complications (Table 3).

**Table 3:** Binary logistic regression of the most prevalent complications by medical specialty

Variable	OR	95% CI	Sig.
Hospital discharge	0.247	(0.33-4.94)	0.72
Accidental removal	0.581	(0.20-1.55)	0.26
Obstruction	0.222	(0.04-14.16)	0.87
MARSI	0.694	(0.13-0.19)	0.32
CLABSI	0.113	(0.110-0.29)	0.85
Thrombosis	0.752	(0.745-4.58)	0.05
No. of lumens	1.662	(0.64-43.11)	0.12
Catheter gauge	0.523	(0.06-5.44)	0.64
Device mean dwell time	0.001	(0.00-1.00)	0.361

* Sociodemographic co-variables included in the analysis, age, gender

## Discussion

The present study found relatively low overall complication rates in PICC devices compared to other studies, particularly in the rates of thrombosis and CLABSI reported elsewhere^13^^, ^^18^. This difference may be attributed to multiple factors, including the experience and training of the professionals involved in PICC care, the expertise of the VAT members, the patient's underlying condition, the adjacent comorbidities, and the pharmacological therapy administered, all of which can influence the variability of PICC complication rates. However, after analyzing the factors associated with patients’ sociodemographic characteristics, our study did not find statistically significant correlations with the prevalence of PICC complications. Therefore, other conditioning factors, such as implementing a VAT staffed by experts in PICC insertion and management, may have contributed to the low complication rates observed in our study^13^^, ^^19^.

This makes it clear the need to reduce variability in PICC management and care by incorporating resources and tools such as ongoing professional training, dissemination of standardized care bundles, and the creation and implementation of VAT experts in PICC insertion and care^9^.

Examining the leading causes of complications observed in our study reveals that PICC accidental removal or dislodgement was the most prevalent, a factor not thoroughly investigated in other studies that should be analyzed in depth due to its significant impact on patients and the associated economic costs for hospitals. This complication may be related to direct elements such as the dressing and securement system used^20^^, ^^22^ and the handling of the device during post-treatment care^23^^, ^^24^. New high-quality studies are recommended to measure this complication, and identify alternative securement methods and the best care routines to reduce its occurrence.

Another complication detected was thrombosis, a complication with a lower prevalence than the reported in other publications^13^ but that continues to present high rates that must be taken into account, especially in hematological patients. Although these patients have a longer PICC dwell time, our study shows that thrombosis is not directly related to catheter size or the mean dwell time. Therefore, it would be important to identify the contributing factors to this difference in future studies.

We observed a higher likelihood of thrombosis associated with the use of multi-lumen catheters, a complication more frequent in hematological patients who require the infusion of multiple substances.

CLABSI, another contributor factor to the high morbidity and mortality rates in hospitalized patients, was rare in our study^14^. These rates align more with those found in studies focused on short-term complications of PICCs^12^.

When comparing our results with previous studies that analyzed complications recorded in the performance of peripherical intravenous catheters (PIVs) placed by a VAT, we found similar rates^9^. However, our study focused especially on onco-hematological patients, who had a longer course of care, allowing monitoring of the devices and recording of their performance until removal. Likewise, conducting further studies that collect prospective information on the catheter care process would be advisable to help identify variables that directly impact complications.

While the need for further studies to gather new data on the direct impact of VATs on patient- perceived quality of care, complication rates of central vascular access devices, and their economic

costs to institutions is acknowledged, in order to make new comparisons on the effectiveness of VATs8, our study makes evident the benefit of a VAT in terms of reducing and controlling complications associated with PICC insertion and management.

## Conclusion

There is a significant decrease in complications, primarily thrombosis and CLABSI, associated with PICCs inserted by a VAT.

Accidental removal is the most prevalent complication, indicating the need to identify its causes and possible alternatives for improvement to reduce its occurrence rate.

Thrombosis is the second most common cause of PICC complications, with catheter gauge and the number of lumens being two variables that contribute to its higher prevalence in hematological patients.

### Implications for nursing

Specialized training for nurses, combined with the practical development of specific skills in the management and care of vascular access devices, leads to better outcomes for critical and complex patients, such as those with onco-hematologic conditions. This expertise helps reduce the number of complications associated with these devices. Consequently, it enhances the quality of patient care and lowers costs related to treating frequent complications like CLABSI and thrombosis.

### Knowledge translation

There is a need to create standardized strategies for unified vascular access care in critical patients to reduce complications.

Establishing specialist teams, such as VATs, has been shown to improve vascular access care, reducing complications.

The prevalence of accidental removal and thrombosis in patients with central vascular access is a key issue that should be addressed in new studies to identify their direct causes.
